# Efficacy of Xuebijing injection on pulmonary ventilation improvement in acute pancreatitis: a systematic review and meta-analysis

**DOI:** 10.3389/fphar.2025.1549419

**Published:** 2025-04-16

**Authors:** Yuling Bin, Rumei Peng, Yaqian Lee, Zhijie Lee, Yang Liu

**Affiliations:** ^1^ Intensive Care Medicine Department of Hengyang Central Hospital, Hunan, China; ^2^ Department of Pathology, Changsha Medical University, Hunan, China

**Keywords:** Xuebijing injection, acute pancreatitis, acute respiratory distress syndrome, respiratory failure, meta-analysis

## Abstract

**Background:**

Xuebijing injection (XBJI), as a Chinese patent medicine injection, consists of five botanical drugs for anti-inflammatory treatment. Acute pancreatitis (AP) is induced by localized inflammation, potentially resulting in multiple organ dysfunction syndromes, specifically including acute lung injury (ALI) and acute respiratory distress syndrome (ARDS). Recent studies suggest that XBJI effective in alleviating potentially easing ALI and ARDS.

**Objective:**

We illustrated the efficacy and safety of XBJI for pulmonary function of AP by conducting a systematic literature review and meta-analysis.

**Methods:**

We conducted searches across eight databases, including PubMed, Embase, and the Cochrane Library, up to September 2024. Two independent investigators screened and selected the literature based on predefined inclusion and exclusion criteria, followed by data extraction. The quality of the selected studies was assessed using the Cochrane Collaboration’s Risk of Bias 2.0 tool. The data were then qualitatively analyzed and synthesized by using Review Manager software, in accordance with the PRISMA guidelines and the Cochrane Handbook.

**Conclusion:**

This study showed that using conventional therapy combined with XBJI might increase the oxygenation index, lower the respiratory rate, and improve APACHE II scores and inflammatory biomarkers. However, there is a high risk of bias and the quality of the included studies is low. More well-designed, large-sample, and high-quality trials are needed to be conducted in multiple centers.

## 1 Introduction

Acute Pancreatitis (AP) is a common cause of acute abdominal pain, and its incidence has been rising globally in recent years due to lifestyle changes and dietary shifts (Boxhoorn et al., 2020). AP is primarily a localized inflammatory condition affecting the pancreas. Approximately 20% of patients often exhibit complications such as systemic inflammatory response syndrome (SIRS) and multiple organ dysfunction syndrome (MODS). The severe complications are accompanied by a mortality rate that ranges approximately from 20% to 40% (Skouras et al., 2014). Among the early complications, acute lung injury (ALI) stands out as one of the most severe, driven by the uncontrolled release of inflammatory mediators, lung tissue inflammation, and oxidative stress. ALI is also prone to developing acute respiratory distress syndrome (ARDS), which could result in respiratory failure (Bhatia and Moochhala, 2004; Schepers et al., 2019). Due to the absence of reliable therapeutic agents, the outcomes for severe AP complicated by respiratory failure remain unsatisfactory, and the mortality rate remains high. Based on the pharmaceutical theory of traditional Chinese medicine, the principal treatment for AP involves clearing heat and expelling evil, replenishing vital energy, nourishing bodily fluids, and promoting blood circulation to dissipate stasis.

Xuebijing injection (XBJI), a standardized traditional Chinese medicine compound, contains five botanical drugs: Honghua, Chishao, Chuanxiong, Danshen, and Danggui (Li et al., 2021; Wu et al., 2021), as summarized in Table 1. It is produced by Tianjin Chase Sun Pharmaceutical Co., Ltd. (China). The functions of XBJI effectively promote blood circulation, remove blood stasis, clear heat, detoxify, and strengthen health (Gong et al., 2024). In recent years, research has demonstrated XBJI has significant efficacy in treating several inflammatory conditions, including sepsis (Wu et al., 2021), severe pneumonia (Guo et al., 2020) and AP (Zhu et al., 2022). The study of Wei Zhou suggest XBJI may modulating the IL-17, TNF, NF-κB, and TLR pathways, and targeting IL-1β, IL-6, and TNF, thereby exert therapeutic effects on sepsis (Zhou et al., 2024). Notably, some preclinical study suggested that XBJI may reduce inflammatory responses, and potentially protect pulmonary function in AP (Chen et al., 2011; Dong et al., 2020; Kang et al., 2010). However, current clinical studies of XBJI protect pulmonary function in AP remain scattered, lacking systematic evidence.

**TABLE 1:** Pharmacological properties of constituent medicinal plants in XBJI.

Scientific name (family name)	Pharmacopoeia name	Parts used	Chinese name	Pharmacological effects
*Carthamus tinctorius* L. (Asteraceae)	Carthami flos	flower	Honghua	Anti-inflammatory, anti-glycemic, anti-thrombotic, anti-cancer (Orgah et al., 2020)
*Paeonia lactiflora* Pall. (Paeoniaceae)	Radix paeoniae rubra	root	Chishao	Anti-inflammatory, anti-viral, anti-bacterial, antioxidant (Parker et al., 2016)
*Ligusticum chuanxiong* Hort. (Apiaceae)	Rhizoma chuanxiong	root	Chuanxiong	Antioxidant, anti-inflammatory, and anti-cancer (Xiang et al., 2022)
*Angelica sinensis* (Oliv.) Diels (Apiaceae)	Radix angelicae sinensis	root	Danggui	Anti-oxidation, promoting immunity, anti-tumor, and anti-bacteria (Wang et al., 2022)
*Salvia miltiorrhiza* Bunge (Lamiaceae)	Salviae miltiorrhizae radix et rhizoma	root	Danshen	Anti-inflammation, anti-oxidation, anti-tumor, anti-atherogenesis, and anti-diabetes (XD et al., 2019)

The aim of this meta-analysis is to systematically evaluate the efficacy of XBJI in protecting pulmonary function in AP. By including randomized controlled trials (RCTs) and clinical controlled trials that meet the inclusion criteria, the analysis will quantitatively assess the impact of XBJI on pulmonary function indicators (e.g., oxygenation index). This meta-analysis aims to provide robust evidence on the efficacy of XBJI for lung protection in AP, thereby supporting both clinical treatment and future research.

## 2 Method

### 2.1 Protocol registration

The study has been officially registered within the International prospective register of systematic reviews (PROSPERO, CRD42024585387).

### 2.2 Literature search strategy

Following the guideline the “Preferred Reporting Items for Systematic Reviews and Meta-analysis (PRISMA)”, We systematically searched multiple databases, including PubMed, Embase, Cochrane Library, Web of Science, Chinese National Knowledge Infrastructure (CNKI), Chinese Biomedical Literature (SinoMed), Wanfang Database, and the China Science and Technology Journal Database, for relevant studies published before September 2024. The search terms included MeSH headings, keywords, abstracts, or titles related to “acute pancreatitis,” “Xuebijing injection,” “acute lung injury,” and “acute respiratory distress syndrome.” There were no language restrictions. Detailed search strategies and the screening process are provided in Supplementary Information 1.

### 2.3 Inclusion criteria

1) Study type: RCTs and clinical controlled trials of XBJI in the treatment of AP.
2) Participants: AP was definitely diagnosed, and the patient nationality, age and gender were unlimited.
3) Intervention measures: The AP patients received conventional treatment, including fluid replacement, correction of water and electrolyte balance, acid-base disorders, gastrointestinal decompression, anti-infection, nutritional support, and inhibition of pancreatic enzyme and the treatment group was supplemented with XBJI on top of conventional treatment.
4) Outcome indicators: main outcome measures: Oxygenation index; Secondary outcome measures: respiratory rate, intra-abdominal pressure, inflammatory factors, APACHE II.

### 2.4 Exclusion criteria

If the above-mentioned conditions are not met, the literature will be excluded. Additionally, studies meeting any of the following criteria will also be excluded.

1) Does not meet the diagnostic criteria for AP.
2) Duplicate literature.
3) Reviews, case reports, and animal experimental studies literature.
4) Intervention measures that are combined with other traditional Chinese medicine treatments.
5) Studies with incomplete data, where attempts to contact the author to obtain complete datasets have failed.

### 2.5 Data extraction process

Two independent investigators (Yuling Bin and Rumei Peng) conducted the study according to the inclusion and exclusion criteria. They separately extracted data that fundamental study details (the publication year, first author), participant characteristics (sample size, age range, gender), intervention measures (treatment duration, dosing schedules), and primary and secondary outcomes.

### 2.6 Quality assessment

The risk of bias in the included studies was independently assessed by the two investigators using the Cochrane Handbook for Systematic Reviews of Interventions and the RoB 2.0 tool. The assessments, conducted in Review Manager 5.3, considered five aspects, like random sequence generation, allocation concealment, blinding, data completeness, and reporting bias. Based on the evaluations, studies are categorized into high, medium, or low quality. Where significant bias risks are identified, sensitivity or subgroup analysis are conducted. Any disagreements that arose during this process were resolved by a third investigator (Yang Liu).

### 2.7 Statistical analysis approach

The statistical analysis of the extracted data was carried out utilizing the Review Manager software. To quantify the combined effect, we transformed continuous outcome data into standardized mean differences (SMDs), accompanied by their respective 95% confidence intervals (CIs). The presence of heterogeneity among the included studies was evaluated using the *I* ^*2*^ test. When an *I* ^*2*^ < 50% indicated the heterogeneity is low, a fixed-effects model was used to make estimates. If not, a random-effects model was applied to statistical analysis. To find the sources of heterogeneity, we conducted subgroup analysis based on the sample size of each group, the duration of treatment, and the publication year.

## 3 Results

### 3.1 Literature retrieves and study characteristics

**FIGURE 1:**
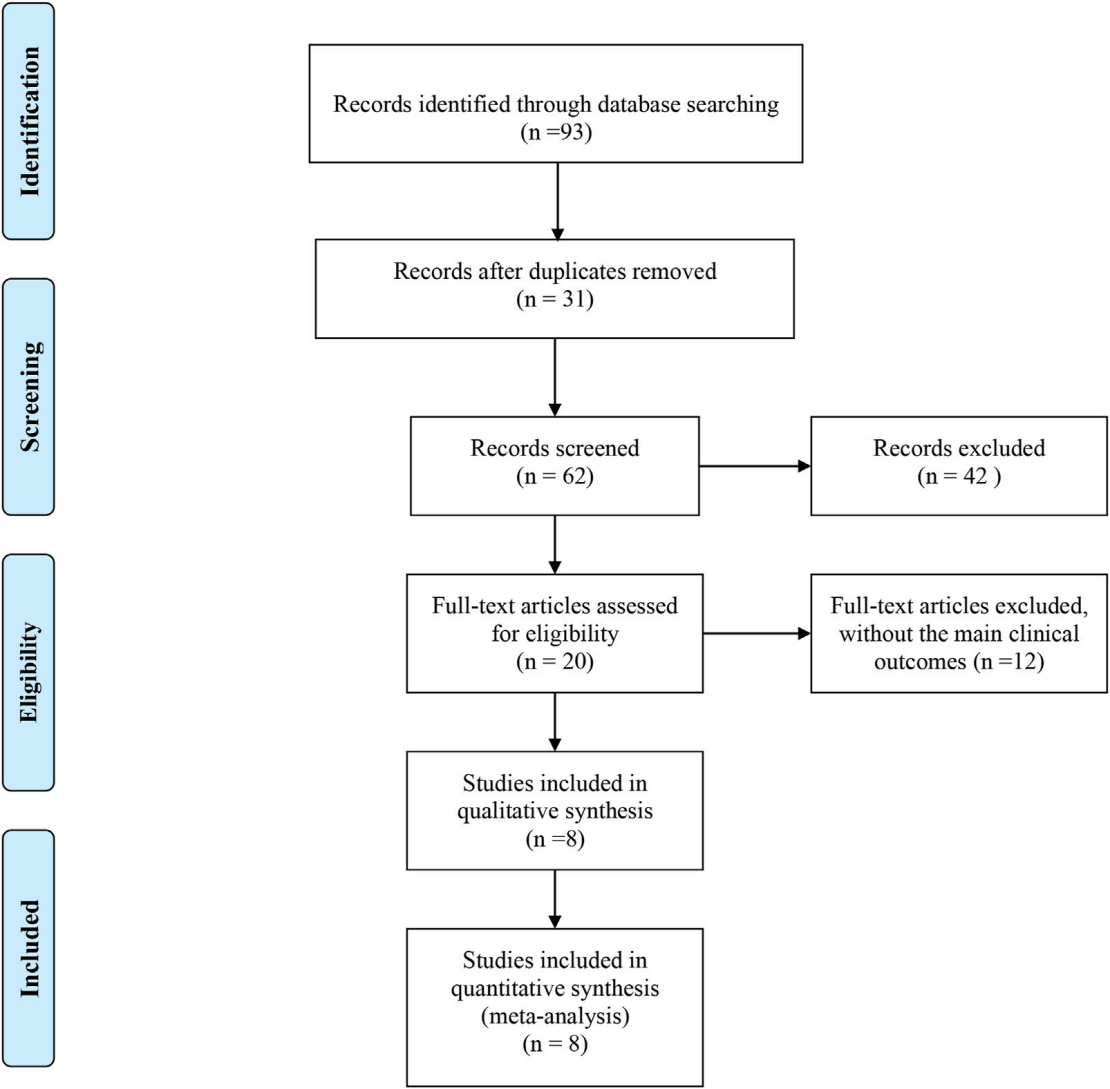
Flow diagram of search methodology and literature selection process.

In this review, we initially retrieved 93 records from eight databases. After removing duplicates and screening the titles and abstracts, we excluded 85 studies due to reasons such as duplication, combination with other interventions, not RCTs or clinical controlled trials, not patients with AP, and the loss of main clinical outcomes. Ultimately, eight articles, encompassing 10 studies with a total of 400 participants, were included in this study (Figure 1). All these studies, published between 2008 and 2022, were carried out in China. The sample sizes ranged from 60 to 144, and the treatment duration was 3–7 days. The control groups received conventional treatment, while the intervention group was supplemented with XJBI. The characteristics of these studies are shown in Table 2.

**TABLE 2:** Characteristics of the included studies.

Study	Number		Sex		Age (years) (mean ± SD)					
	T	C	M	F	T (XBJ)	C	Duration	Treatment	Control	Outcomes
(Feng Yan, Wang Jianhua, Zhu June 2008)	34	34	42	26	43		7 days	XBJI 100 mL ivgtt bid + CT	CT	①,②
(Chenkunshun 2011)	30	30	34	26	45.3 ± 12.1		7 days	XBJI 100 mL ivgtt Q8h + VT + CT	CT	①
(Wangjue and Liruhong 2011)	30	30	35	25	52.3 ± 9.5	50.1 ± 10.7	7 days	XBJI 100 mL ivgtt bid + CT	CT	①,②,⑤
(Wangjue and Liruhong 2011)	30	30	35	35	+UST:50.3 ± 10.5	51.3 ± 11.0		XBJI 100 mL ivgtt bid + UST 200,000 IU Q12h + CT	CT + UST	
(Chenjialian et al., 2018)	54	54	57	51	44.21 ± 9.62	44.16 ± 9.52	7 days	XBJI 100 mL ivgtt bid + CT	CT	①,⑤
(Chang Xiao et al., 2019)	30	30	34	26	53. 6 ± 7. 9	51. 3 ± 7. 2	7 days	XBJI 50 mL ivgtt bid + CT	CT	①,③,④
(Fanhai, 2019)	40	40	51	29	42.4 ± 11.7	41.7 ± 12.2	7 days	XBJI 10 mL ivgtt bid + CT	CT	①,③,④
(Liu and Qu 2020)	72	72	83	61	43.62 ± 8.53	43.25 ± 8.37	7 days	XBJI 10 mL ivgtt bid + CT	CT	①,④
(Ge Kaijie and Meng, 2022)	40	40	98	62	54.12 ± 2.07	54.24 ± 6.52	3 days	XBJI 100 mL ivgtt bid + CT	CT	①,④,⑤
(Ge Kaijie and Meng, 2022)	40	40			53.69 ± 1.93s			XBJI100 mL ivgtt Q8h + CT		

UST, ulinastatin; XBJI, xuebijing injection; NR, no related; T, treatment; C, control; SD, standard error; M, man; F, female; CT, conventional therapy; NS, normal saline.

Oxygenation index ①, respiratory rate ②, intra-abdominal pressure ③, inflammatory factors ④, APACHE II ⑤.

### 3.2 Meta-analysis results of the main outcome indicators

#### 3.2.1 Oxygenation index

**FIGURE 2:**
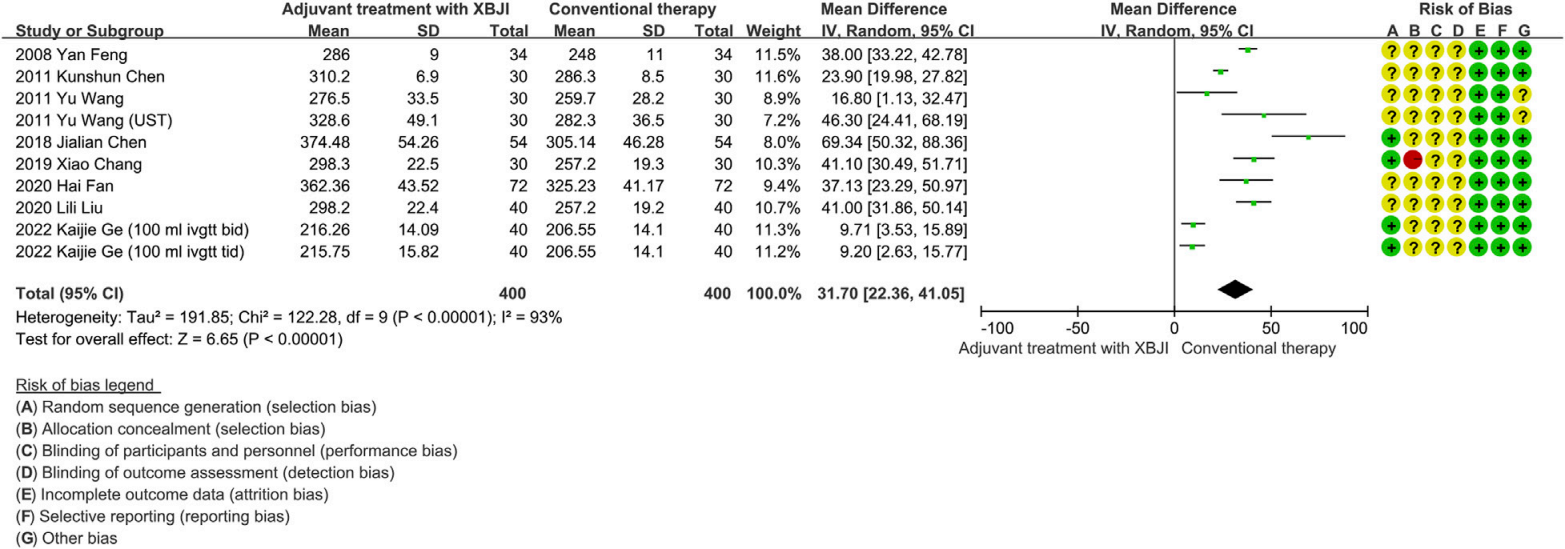
The meta-analysis results revealed that the combination of conventional therapy and XBJI significantly improved the oxygenation index in AP patients (MD = 31.70, 95% CI [22.36, 41.05], *P* < 0.00001).

Ten studies have reported on the oxygenation index of patients with AP following both conventional therapy and a combined treatment utilizing XBJI. Heterogeneity tests conducted on these studies revealed a high degree of variability (*P* < 0.00001, *I* ^*2*^ = 93%). A sensitivity analysis was subsequently performed, which demonstrated that the exclusion of any individual study did not significantly alter the results, suggesting their stability despite the heterogeneity. Despite analyzing the clinical, methodological, and statistical heterogeneity across these studies, the specific source of this variability remained unclear. Consequently, a random effects model was employed for the meta-analysis. The findings indicated a statistically significant difference (MD = 31.70, 95% CI [22.36, 41.05], *P* < 0.00001), demonstrating that the combination of XBJI with conventional therapy was more effective in improving the oxygenation index of patients with AP compared to conventional therapy alone (Figure 2).

### 3.3 Meta-analysis results of the secondary outcome indicators

#### 3.3.1 Respiratory rate

**FIGURE 3:**
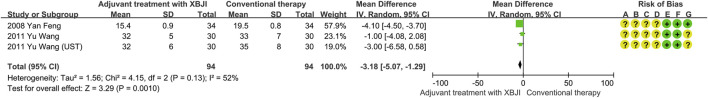
The meta-analysis results revealed that the combination of conventional therapy and XBJI significantly decreased the respiratory rate in AP patients (MD = −3.18, 95%CI [-5.07, 1.29], *P* = 0.0010).

Three studies employed respiratory rate as a secondary outcome indicator, encompassing 94 participants in a combined treatment utilizing XBJI and conventional therapy. Given the substantial heterogeneity observed (*P =* 0.13, *I* ^*2*^ = 52%), the random effects model was utilized for data evaluation. The results of the meta-analysis revealed a statistically significant advantage for the combined treatment group in decreasing respiratory rate compared to the conventional therapy group (MD = −3.18, 95%CI [-5.07, 1.29], *P <* 0.00001) (Figure 3).

#### 3.3.2 Intra-abdominal pressure

**FIGURE 4:**
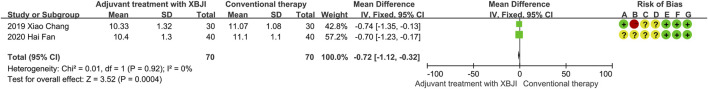
The meta-analysis results revealed that the combination of conventional therapy and XBJI significantly reduced the intra-abdominal pressure levels in AP patients (MD = −0.72, 95%CI [-1.12, −0.32], *P* = 0.0004).

A total of two studies reported on intra-abdominal pressure levels, encompassing 70 cases in a combined XBJI treatment group and a conventional therapy group. Given the low heterogeneity observed (*P* = 0.92, *I* ^*2*^ = 0%), fixed effect models were employed. Meta-analysis revealed that the combined XBJI treatment group exhibited a significant reduction in intra-abdominal pressure compared to the conventional therapy (MD = −0.72, 95%CI [-1.12, −0.32], *P* = 0.004) (Figure 4).

#### 3.3.3 IL-6

**FIGURE 5:**
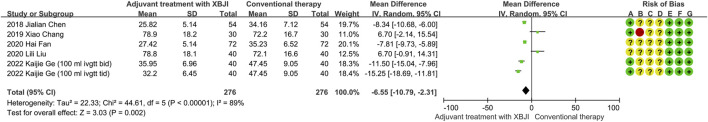
The meta-analysis results revealed that the combination of conventional therapy and XBJI significantly reduced the IL-6 in AP patients (MD = -6.55, 95%CI [-10.79, -2.31], *P* = 0.002).

Six studies employed IL-6 as an outcome measure, with 276 participants in both the adjuvant treatment with XBJI and conventional therapy. Given the significant heterogeneity observed (*P* < 0.00001, *I* ^*2*^ = 89%), the random effects model was utilized for data evaluation. The meta-analysis results revealed a notable superiority of the adjuvant treatment with XBJI in reducing IL-6 levels compared to the conventional therapy (MD = −6.55, 95%CI [-10.79, −2.31], *P* < 0.00001) (Figure 5).

#### 3.3.4 TNF-α

**FIGURE 6:**
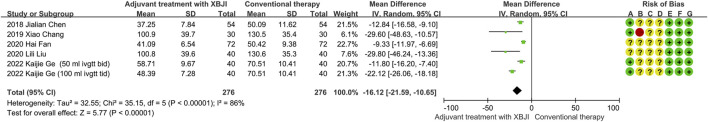
The meta-analysis results revealed that the combination of conventional therapy and XBJI significantly reduced the TNF-α in AP patients (MD = −16.12, 95%CI [-21.59, −10.65], *P* < 0.00001).

Six studies utilized TNF-α as one of the outcome indicators. Due to the substantial heterogeneity observed (*P* < 0.00001, *I* ^*2*^ = 86%), the random effects models were employed for analysis. The meta-analysis results demonstrated that the adjuvant treatment with XBJI exhibited a significant advantage over the conventional therapy in reducing TNF-α levels (MD = −16.12, 95%CI [-21.59, −10.65], *P* < 0.00001) (Figure 6).

#### 3.3.5 APACHE II

**FIGURE 7:**
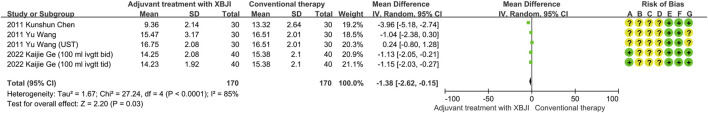
The meta-analysis results revealed that the combination of conventional therapy and XBJI significantly decreased the APACHE II score in AP patients (MD = −1.38, 95% CI [−2.62, −0.15], *P* = 0.03).

Five studies assessed APACHEII score. Compared with conventional therapy, the level of APACHEII score was significantly decreased in the adjuvant treatment with XBJI group (MD = −1.38, 95% CI [−2.62, −0.15], *P* < 0.00001) (Figure 7), without significant heterogeneity (*I* ^2^ = 85%, *P* < 0.00001).

### 3.4 Trial sequential analysis

**FIGURE 8:**
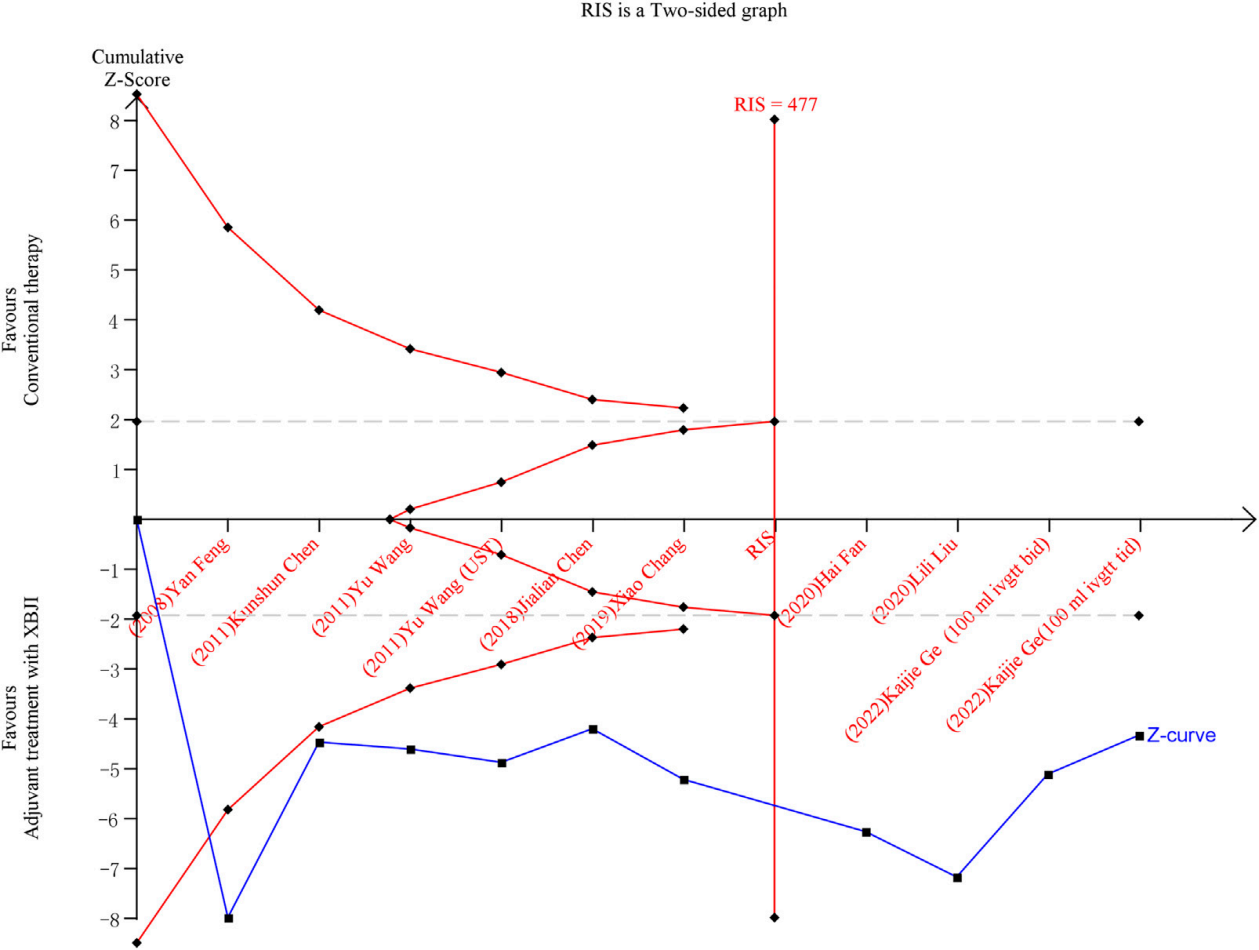
TSA result of the oxygenation index after conventional therapy and combination of XBJI in AP patients. The cumulative Z-score (depicted in blue), representing the accumulated level of statistical significance, was calculated after each new trial was included. The grey lines denote traditional boundaries. The oblique red dashed lines symbolize the trial sequential monitoring boundaries and the futility boundaries. The vertical red dashed line signifies the estimated RIS.

Ten included studies were conducted on the oxygenation index using TSA software. Following the inclusion of the first study, the cumulative Z-value crossed the conventional threshold (Z = 1.96). After the inclusion of the sixth study, the sample size exceeded the RIS (Required information size), indicating that the combination of XBJI with conventional treatment enhances the oxygenation index in AP patients, and the required number of cases has been reached (Figure 8).

## 4 Discussion

### 4.1 Principal findings

In recent decades, researches on AP have primarily concentrated on trypsinogen activation, malfunctions in pancreatic microcirculation, calcium overload, and inflammatory pathways (Mihoc et al., 2024). However, the edema of peripancreatic tissue, additional fluid resuscitation further increase IAP, sever AP patients developing ALI and ARDS leading to up to 60% of the mortality occurring in the first week of the disease (Jacobs et al., 1977; Sah et al., 2013). Due to the available treatment of AP are limited, leading researchers focus on complementary and alternative therapies. The traditional Chinese medicine always has demonstrated its superiority in the management of AP and other inflammatory diseases for centuries in China (Li et al., 2017).

XBJI has been employed as an adjuvant therapy in the treatment of sepsis, severe pneumonia, and AP. XBJI appears to be a multifaceted mechanism encompassing anti-inflammatory effects, antioxidant properties, and immune modulation. It exhibits the capability to suppress the excessive secretion of inflammatory mediators, thereby mitigating pulmonary inflammatory responses (Zhang et al., 2022). Simultaneously, its antioxidant constituents effectively scavenge free radicals, alleviating the detrimental impact of oxidative stress on lung tissue. Furthermore, by modulating the function of immune cells, XBJI may also facilitate the repair and regeneration of lung tissue, further enhancing its protective efficacy (Lv et al., 2022).

In previous meta-analyses, the application of XBJI has encompassed a broad range of diseases. For instance, Zhang et al. performed a meta-analysis demonstrating adjuvant therapy of increased the total effectiveness rate of AP patients shortened the abdominal pain, distension relief time and reduced serum amylase level (Zhu et al., 2022). Yun Zhang’s meta-analysis confirms that XBJI exhibits therapeutic effects on ARDS patients, partly through the regulation of immune cell and cytokine pathways. It inhibits TNF-α, while exerting an indirect suppressive effect on IL-6 (Zhang et al., 2021). The meta-analysis conducted by Chengyu Li showed XBJ adjuvant therapy could potentially improve sepsis outcomes by decreasing the 28-day mortality rate, APACHE II scores, and WBC count (Li et al., 2018). These findings reinforce our results, underscoring the potential benefits of XBJI across various conditions. It has shown unique advantages in targeting complex conditions and saving the lives of patients. Collectively, XBJI could play an important and expanding role in clinical medicine, especially for AP, severe pneumonia and sepsis. However, the impact of XBJI on improving pulmonary ventilation, specifically in the AP, has not been inadequately evaluated. This meta-analysis complements the deficiencies of previous research, providing evidence-based medical support for the widespread clinical application of XBJI.

### 4.2 Limitations and future perspectives

The studies included in the meta-analysis remain inadequate. Firstly, the analysis lacks data on the primary causes of AP patients, potentially hindering an accurate assessment of the therapeutic efficacy of XBJI. Secondly, the number of studies included are limited, and the quality of these studies are uneven, from single-center studies with small sample sizes. AP Patients from diverse hospitals may display certain heterogeneity. Furthermore, the AP patients included in the studies were not classified based on the severity of their disease. Furthermore, the duration of treatment across studies were inconsistent, potentially introducing bias into the research outcomes. Chronic complications of AP, including pancreatic pseudocysts, chronic pancreatitis, and pancreatic cancer, unfortunately, were not adequately addressed with follow-up data in these studies.

XBJI was approved in the Chinese market for nearly 2 decades. The adverse drug reactions observed with XBJI included skin pruritus, erythema, and chest tightness. These reactions were often linked to the irrigation process, specifically when the syringe fluid was administered too rapidly. Most reactions were relatively mild or non-serious (Zheng et al., 2019). The overall incidence of allergic reactions tended to escalate with both increased dosage and patient age. Furthermore, when XBJI was combined with other drugs, such as Ringer’s sodium acetate solution, reduced glutathione, aspirin-DL-lysine, and torasemide, the risk of adverse reactions heightened (Wang et al., 2019). There remains a notable absence of international standards for assessing its safety and efficacy. Jie Yang et al. demonstrated target trial emulation principles, presenting replicable protocols for eligibility, intervention standardization, and outcome evaluation in observational studies. This may establish the framework for XBJI trials (Yang et al., 2025). Additionally, our findings should be interpreted with caution, as critical methodological details (e.g., randomization procedures, blinding) were unreported in the primary studies. To address the issue of unknown bias risk, it is necessary to provide detailed information on study design, sample selection, data collection, and analysis methods in traditional Chinese medicine studies. Consequently, more preclinical studies and multi-center prospective cohort studies, with significantly larger sample sizes need be undertaken, in order to validate and strengthen these findings.

## 5 Conclusion

In this meta-analysis, the results indicated that combination of conventional therapy and XBJI, when compared to conventional therapy, exhibits favorable effects in enhancing the oxygenation index, decreasing respiratory rate, and improving APACH II scores, inflammatory biomarkers such as IL-6 and TNF-α. Notably, a significant heterogeneity was observed in the oxygenation index (*I* ^*2*^ = 89%). To address this issue, we carried out a subgroup analysis based on patient number, XBJI dosage and the duration of treatment. Unfortunately, despite these efforts, the heterogeneity among studies did not decrease significantly. Consequently, we resorted to utilizing a random effects model to pool the statistical measures. In summary, the combination of XBJI with conventional treatment demonstrates certain advantages in enhancing the oxygenation index, decreasing respiratory rate and intra-abdominal pressure, as well as improving certain inflammatory indicators.

## Abbreviations

XBJI, Xuebijing injection; AP, Acute pancreatitis; ALI, acute lung injury; ARDS, acute respiratory distress syndrome; SIRS, systemic inflammatory response syndrome; MODS, multiple organ dysfunction syndrome; CNKI, Chinese National Knowledge Infrastructure; PRISMA, Systematic Reviews and Meta-analysis; SinoMed, Chinese Biomedical Literature; UST, Ulinastatin; NR, No related; T, Treatment; SD, Standard error; M, Man; F, Female; C, Control; CT, Conventional therapy; NS, normal saline; TSA, Trial sequential analysis; RIS, Required Information Size.
