# Supplementary material for: Efficacy of Xuebijing injection on pulmonary ventilation improvement in acute pancreatitis: a systematic review and meta-analysis
Source: Front Pharmacol. 2025 Apr 16;16:1549419. doi: 10.3389/fphar.2025.1549419 (PMC12041077; doi:10.3389/fphar.2025.1549419)
Supplement: Supplementary file 1 [file Table1.docx]

**Literature search**

**PubMed**

(("acute pancreatitis"[Title/Abstract]) OR (((Pancreatitis[MeSH Terms]) OR (pancreatitis[Title/Abstract])) AND (("Acute Disease"[MeSH Terms]) OR ("acute*"[Text Word])))) AND (("xuebijing injection"[Text Word]) OR (xuebijing[Text Word]))

**Embase**

#1. 'pancreatitis'/exp OR pancreatiti*:ti,ab,kw OR 'pancreatic next1/1 inflamm*':ab,ti

#2. 'xuebijing':ti,ab,kw OR 'xuebijing injection':ti,ab,kw

#3. 'acute lung injury' OR (acute AND ('lung'/exp OR 481,984

lung) AND ('injury'/exp OR injury)) OR 'acute

lung injury'/exp OR ali OR ards OR 'ards'/exp OR

'acute respiratory distress syndrome'/exp OR

'acute respiratory distress syndrome' OR (acute

AND ('respiratory'/exp OR respiratory) AND

('distress'/exp OR distress) AND ('syndrome'/exp

OR syndrome))

#4. #1 AND #2 AND #3

**Web of Science**

1: "acute pancreatitis" (Topic) OR ("Pancreatitis" OR "pancreatitis") AND ("Acute Disease" OR "acute*") (Topic) Results: 36160

2: "acute lung injury" (Topic) OR ALI (Topic) OR ARDS (Topic) OR " acute respiratory distress syndrome" (Topic) Results: 69864

3: "xuebijing injection" (Topic) OR "xuebijing" (Topic) Results: 284

4: #3 AND #2 AND #1 Results: 0

5: #3 AND #1 Results: 5

**Cochrane**

#1 (acute pancreatitis):ti,ab,kw OR (pancreatitis):ti,ab,kw OR (pancreatitis, acute hemorrhagic):ti,ab,kw (Word variations have been searched)

#2 ("xue-bi-jing"):ti,ab,kw OR ("xue bi jing"):ti,ab,kw OR ("xuebijing"):ti,ab,kw OR ("xuebijing injection"):ti,ab,kw (Word variations have been searched)

#3 #1 AND #2

知网

SU=('血必净'+'血必净注射液') AND SU=('胰腺炎'+'急性胰腺炎') AND SU=('呼吸衰竭'+'急性呼吸窘迫症'+'急性肺损伤'+'ALI'+'ARDS')

SinoMed database

1）"急性胰腺炎"[常用字段:智能] OR "胰腺炎"[常用字段:智能] 140840

2) "血必净"[常用字段:智能] OR "血必净注射液"[常用字段:智能] 3577

4) "呼吸衰竭"[常用字段:智能] OR "急性肺损伤"[常用字段:智能] OR "急性呼吸窘迫症"[常用字段:智能] OR "ALI" [常用字段:智能] OR "ARDS"[常用字段:智能] 217913

5) ("呼吸衰竭"[常用字段:智能] OR "急性肺损伤"[常用字段:智能] OR "急性呼吸窘迫症"[常用字段:智能] OR "ALI" [常用字段:智能] OR "ARDS"[常用字段:智能]) AND ("血必净"[常用字段:智能] OR "血必净注射液"[常用字段:智能]) AND ("急性胰腺炎"[常用字段:智能] OR "胰腺炎"[常用字段:智能]) 10

VIP

U=(急性胰腺炎+胰腺炎) AND U= (血必净+血必净注射液) AND U= (呼吸衰竭+急性肺损伤+急性呼吸窘迫症+ALI+ARDS)

万方

主题:(“胰腺炎” or “急性胰腺炎” ) and 主题: (“血必净” or “血必净注射液”) and 主题: (“呼吸衰竭” or “急性肺损伤”or "急性呼吸窘迫症" or "ALI" or "ARDS")
